# Patterns and correlates of casual sexual behavior through the internet and inconsistent condom use among MSM college students in Zhejiang province, China: a cross-sectional study, 2022

**DOI:** 10.3389/fpubh.2026.1860278

**Published:** 2026-07-20

**Authors:** Lin Chen, Jun Jiang, Yun Xu, Xin Zhou, Jiezhe Yang, Xiaohong Pan, Wei Cheng, Chengliang Chai, Jianmin Jiang

**Affiliations:** 1 Department of HIV/AIDS and STDs Control and Prevention, Zhejiang Provincial Center for Disease Control and Prevention, Hangzhou, Zhejiang, China; 2 Zhejiang Key Lab of Vaccine, Infectious Disease Prevention and Control, Zhejiang Provincial Center for Disease Control and Prevention, Hangzhou, Zhejiang, China

**Keywords:** casual sexual behavior, cross-sectional study, human immunodeficiency virus (HIV), internet, men who have sex with men

## Abstract

**Objectives:**

To identify factors associated with casual sexual behavior mediated via the Internet (CSBI) and condom use with casual sexual partners found on the Internet (CSPI) among men who have sex with men (MSM) college students in Zhejiang Province, China, we conducted a cross-sectional study in six cities of Zhejiang in 2022.

**Methods:**

All subjects were recruited by four community-based organizations (CBOs), and information on demographic characteristics, risky sexual behavior, and health practices was collected. Univariate and multivariable logistic regression analyses were performed using SPSS software to identify factors associated with CSBI and condom use with CSPI.

**Results:**

Of the 671 participants, 73.3% (492/671) identified as gay, 317 (47.2%) reported CSBI in the past 6 months, and 31.5% (100/317) of these reported inconsistent condom use with these CSPI. The odds of engaging in CSBI in the past 6 months were significantly higher among MSM who watched pornographic videos ≥3 times per month (adjusted Odds Ratio (aOR) = 2.404, 95% confidence interval (CI): 1.598–3.617), and those who had a regular male sexual partner (aOR = 1.900, 95% CI:1.369–2.637). The odds of inconsistent condom use with CSPI in the past 6 months were significantly higher among participants who disclosed sexual orientation (aOR = 1.711, 95% CI: 1.010–2.898), those identified as bisexual or uncertain sexual orientation (aOR = 2.228, 95% CI: 1.241–3.999), and those had sexual contact with “both student and non-student partners” (aOR = 1.897, 95% CI: 1.092–3.297). In contrast, participants with a high level of condom use self-efficacy (aOR = 0.340, 95% CI: 0.212–0.657) and those who obtained human immunodeficiency virus (HIV) knowledge from the MSM communities (aOR = 0.447, 95% CI: 0.245–0.815) had a significantly lower odds of inconsistent condom use.

**Conclusion:**

A high prevalence of web-based casual sexual behavior was observed among MSM college students, particularly among those who frequently viewed pornography or maintained a stable male sexual partner. Students who have sexual contact with non-student partners were associated with inconsistent condom use. Additionally, MSM community-based interventions need more support to improve HIV knowledge and condom use with online casual partners.

## Introduction

Globally, men who have sex with men (MSM) are the group at high risk of human immunodeficiency virus (HIV) infection, with a prevalence of 5–30%; this is higher than that in the general population (1, 2). In China, male–male sexual contact is an important route of HIV transmission, accounting for nearly 30% of newly diagnosed cases in recent years (3). Zhejiang Province in southeastern China, has a low overall HIV prevalence but a high burden of HIV infection among MSM. MSM accounted for 40% of all HIV patients newly diagnosed over the past 5 years, with a 4–8% prevalence in this subgroup. MSM college students reported a 2–4% HIV positive rate, which was approximately 100 times higher than that of the general male student population (4).

The advent of the Internet era and the spread of “social applications” may be changing the mode of HIV transmission among both MSM and MSM college students (5, 6). “Blued” is the most popular social app among MSM, with more than 40 million registered users in China and 409,000 in Zhejiang (7). Casual sexual behavior via the Internet (CSBI) and condom use with casual sexual partners found on the Internet (CSPI) are key determinants of HIV transmission. The prevalence of CSBI among MSM ranges from 29 to 90% across different countries and is increasing annually worldwide (8, 9). Convenience, accessibility, and anonymity are important reasons for the prevalence of online sexual partner-seeking behaviors. However, multiple sexual partners and unawareness of partner’s HIV status during online hookups elevate the risk of HIV transmission (5).

The MSM who engage in CSBI are more likely to report risky behaviors, such as low perceived risk, substance abuse during sex, multiple sexual partners, and more sexually transmitted diseases (10–12). MSM college students also engage in such risky behaviors. A study of 447 MSM college students in China revealed that nearly all of them used social apps, and those who used these apps to seek sexual partners were more likely to engage in high-risk sexual behaviors, including having multiple sexual partners, engaging in group sex, participating in commercial sex, and using recreational drugs during sex (13).

In the context of the rapid development of the internet, MSM college students have faced new challenges, such as the spread of stigma online, increased loneliness, depression, and anxiety, and the proliferation of pornography (14, 15). Few studies have examined the characteristics and correlates of CSBI and condom use with CSPI among MSM college students, with a paucity of studies performed integrated analyses from sociological, behavioral and epidemiological perspectives.

The HIV transmission from MSM to heterosexuals is another issue meriting attention. Men who have sex with both men and women have a prevalence of sexually transmitted infections (STIs) that is similar to or even higher than that of MSM only (16, 17). Bisexual behavior is common among MSM college students but rarely reported truthfully, while self-identified bisexual orientation is an important predictor of such behavior.

To further understand the characteristics of HIV-related behaviors among MSM college students, we conducted a cross-sectional study to explore the status of CSBI and compared the risky behaviors between gay men and bisexual/uncertain groups. We also explored whether sexual orientation, HIV knowledge and access, post-exposure prophylaxis (PEP), pre-exposure prophylaxis (PrEP), and HIV testing were associated with CSBI and inconsistent condom use.

## Materials and methods

### Study population

This cross-sectional study enrolled MSM college students from March 28, 2022, to July 31, 2022 in Zhejiang Province. The enrollment criteria were as follows: (1) male college students aged 18 years or older; (2) having ever engaged in anal or oral intercourse with another male; (3) self-identified as gay, bisexual, or of uncertain sexual orientation; (4) providing informed consent to participate in the study.

### Study design and data collection

Some MSM students could be contacted by community-based organizations (CBOs) on dating applications or social platforms. Most of them were active online but difficult to reach and recruit for research purposes. This study adopted a modified respondent-driven sampling (RDS) method for recruitment.

MSM college students were enrolled by four local CBOs in six cities (Hangzhou, Ningbo, Jinhua, Jiaxing, Shaoxing, and Wenzhou), which serve more than 70% of all MSM in Zhejiang. First, recruitment advertisements were posted on social applications (e.g., WeChat, QQ, Blued, Finka, Aloha, Zank), social platforms (WeChat Official Accounts, Douyin Official Accounts, and others related platforms), and in CBOs’ studios from March and July 2022. Second, students recruited by CBOs were encouraged to recommend as many of their MSM friends as possible to participate in this study.

The CBOs were responsible for conducting an investigation in their studios. All participants were instructed to scan a two-dimensional code using their mobile devices, authorize the use of their phone numbers, and proceed to the electronic informed consent form (eICF). After completing the eICF, participants gained access to the electronic questionnaire. The electronic questionnaire, comprising 60 items (48 of which were mandatory), was pretested with 30 participants to ensure item clarity and validity. Respondents could review and revise their previous answers via the built-in back button during the survey.

**Figure 1:**
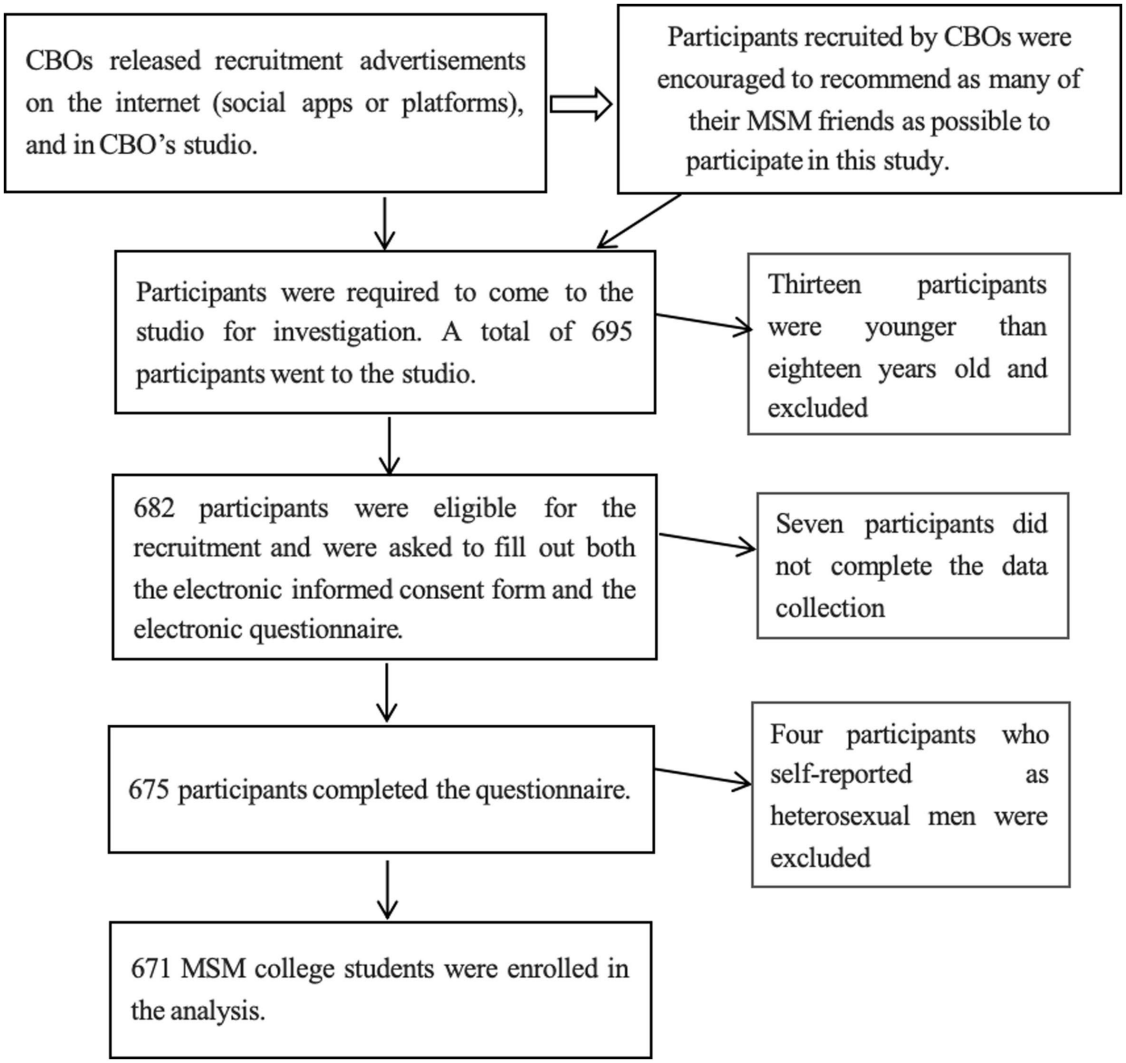
Recruitment process flow chart for MSM college students. The figure shows the recruitment process and results of MSM college students in the study.

A face-to-face training session was provided by CBOs to clarify the definitions of specific survey items. CBO’s staff checked the questionnaires and corrected missing or incorrect information after contacting participants by phone every day. Following verification of phone numbers, no duplicate registrations were identified. The recruitment process diagram is illustrated in Figure 1.

The sample size was calculated based on the rate of seeking sexual partners found online among MSM in China, which ranged from 40 to 60% (5, 6). According to the formula for cross-sectional surveys, the minimum sample size for this study was estimated at 600 participants, with an *α* = 0.05 and *β* o = 0.1.

$$N=400*\frac{1-p}{p}$$

### Related definition

The following question asked about CSBI in the past 6 months: *“Have you ever had sexual intercourse (oral or anal) with men you met online, such as through Blued, WeChat, a chat room, or other sites in the past 6 months?”* Another question evaluated inconsistent condom use with CSPI: *“How often did you use condoms with online casual sexual partners during anal sex in the past 6 months?”* The response options were “Every time, Mostly, Rarely, and Never.” Inconsistent condom use was defined as selecting “Mostly, Rarely, or Never” in response to this question. Stimulant use was evaluated with the following question: “*Do you ever use any stimulants (including various nitrite esters, e.g., ‘Rush poppers’, ‘No. 0 capsule’) with your most recent casual sexual partners found online?*”

One question was used to evaluate the source of HIV knowledge among MSM individuals: “*Does your HIV knowledge mainly come from the MSM community?”* Sexual orientation was assessed using the question *“How would you identify your sexual orientation: Gay, Bisexual, Heterosexual, Uncertain?.”* Disclosed sexual orientation was determined by the question *“Have you ever told anyone about your sexual orientation?”*

Five questions were designed to evaluate HIV knowledge level, as shown in Table 1. A score of 1 point was assigned for a correct answer and zero points for incorrect answer.

**Table 1:** Definition of HIV knowledge and condom use self-efficacy.

Title	Options
Five HIV knowledge questions	
*① Men who have sex with men are the most severely affected by HIV in China.*	*Correct (answer)/Incorrect/Do not know*
*② Occasionally condom non-use will not result in HIV infection.*	*Correct/Incorrect (answer)/Do not know*
*③ Post-exposure prophylaxis (PEP) can be administered within 96 h after high-risk behavior, with optimal efficacy if taken within 2 h.*	*Correct/Incorrect (answer)/Do not know*
*④ Which of the following anal sexual roles carries a higher risk of HIV infection?*	*Insertive/Receptive (answer)/Equal risk/Unsure*
*⑤ Stimulant use during sexual activity increases the risk of HIV infection.*	*Correct (answer)/Incorrect/Do not know*
Condom use self-efficacy questions	
*① Do you have confidence in discussing condom use prior to sexual intercourse with casual partners met through online dating platforms or apps?*	*Scoring:1(Strongly disagree) to 5(Strongly agree)*
*② Do you have confidence in refusing sexual activity if your casual partner met through online dating platforms or apps asks you not to use a condom?*	*Scoring:1(Strongly disagree) to 5(Strongly agree)*
*③ Do you have confidence in preparing condoms in advance before seeking casual sexual partners online?*	*Scoring:1(Strongly disagree) to 5(Strongly agree)*

Three questions were used to assess condom use self-efficacy, as shown in Table 1. A score of ≥9 points was classified as high, while a score of <9 points was classified as low; the lower the score, the lower the corresponding confidence level.

### Ethical considerations

The eICF provided two response options: “*Agree*” and “*Disagree*”. If participants selected *“Agree,”* they were granted access to the electronic questionnaire to complete it. If they selected *“Disagree,”* the survey was automatically terminated with a corresponding prompt. All participants included in the final analysis had selected the *“Agree”* option. The eICF stipulated that de-identified data could be used for secondary analysis without the need for re-consent. Furthermore, the document clarified that although the survey was conducted anonymously, participants were required to provide their mobile phone numbers for duplicate entry detection. All participants authorized the use of their contact information via secure digital channels. Participants received a USD 5 research incentive distributed by CBOs, while researchers received a USD 10 honorarium per participant for their involvement in study administration.

### Statistical analysis

SPSS version 25.0 was used for data analysis. Descriptive analyses were performed to characterize the demographic features of MSM college students, including those who engaged in CSBI. The chi-square test was applied to compare the characteristics of CSBI among those stratified by sexual orientation. Univariate and multivariable logistic regression analyses (using the purposeful selection method) were conducted to calculate crude odds ratios (OR), adjusted odds ratios (aOR), and 95% confidence intervals (CI) for two primary outcomes: casual sexual behaviors via the Internet and inconsistent condom use with CSPI in the past 6 months (18).

All variables with a *p-value less than* 0.2 in the univariate analysis were entered into the multivariable model. Furthermore, extra covariates were incorporated into the multivariable analysis based on relevant literature or research experience: age, sexual orientation (for the outcomes of CSBI); age, anal sex role with CSPI, and PEP uptake (for the outcome of inconsistent condom use with CSPI in the past 6 months) (5, 6). A *p-*value less than 0.05 was considered statistically significant. Missing data (from 18 participants who did not respond to the question on HIV self-testing) were explicitly stated in the table footnotes and excluded from all statistical analyses.

## Results

### Demographic characteristics

The demographic assessments included 671 MSM college students, among whom 65.9% (442/671) were registered in Zhejiang, 69.3% (465/671) had a bachelor’s degree or higher, and 67.8% (455/671) were 21 years old or older. One hundred eighty-one (27.1%) reported living expenses ≧ 2,001 CNY per month. Of the subjects, 73.3% (492/671) self-identified as gay, 20.3% (136/671) as bisexual, and 6.4% (43/671) as being of uncertain sexual orientation (Table 2).

**Table 2:** Demographic characteristics of the MSM college students and those who engage in CSBI in the past 6 months.

Variables	All (*n* = 671) *n* (%)	CSBI in the past 6 months	
		Yes (*n* = 317) *n* (%)	No (*n* = 354) *n* (%)
Age, years			
18–20	218 (32.2)	90 (28.4)	126 (35.6)
21–23	358 (53.4)	179 (56.5)	179 (50.6)
≥24	97 (14.5)	48 (15.1)	49 (13.8)
Registered permanent residence			
Zhejiang Province	442 (65.9)	197 (62.1)	245 (69.2)
Other Provinces	229 (34.1)	120 (37.9)	109 (30.8)
Educational level			
Junior college	206 (30.7)	94 (29.7)	112 (31.6)
Bachelor’s degree or above	465 (69.3)	223 (70.3)	242 (68.4)
Academic year			
Freshman or sophomore	360 (53.7)	162 (51.1)	198 (55.9)
Junior, senior or postgraduate	311 (46.3)	155 (48.9)	156 (44.1)
Monthly living expenses (CNY)			
0–1,500	209 (31.3)	109 (28.2)	100 (34.7)
1,501–2,000	278 (41.6)	128 (42.4)	254 (40.8)
≥2,001	181 (27.1)	77 (29.4)	77 (24.5)
Sexual orientation			
Gay	492 (73.3)	236 (74.4)	256 (72.3)
Bisexual	136 (20.3)	68 (21.5)	68 (19.2)
Uncertain	43 (6.4)	13 (4.1)	30 (8.5)

### Associated factors of seeking casual sexual partners on the internet

Among 671 MSM college students, 317 (47.2%) reported CSBI in the past 6 months. Univariate and multivariable regression models demonstrated that students who watched pornography three or more times per month (aOR = 2.404, 95% CI: 1.598–3.617) had significantly higher odds of engaging in CSBI compared with those who watched pornography less than once per month. Students with a stable male sexual partner were 1.900 times more likely to seek sexual partners online than those without such a partner (95% CI: 1.369–2.637). Additionally, participants who used PEP previously were more likely to engage in CSBI than those who did not (aOR = 2.154, 95% CI: 1.220–3.802) (Table 3).

**Table 3:** Univariate and multivariable logistic regression analyses for predictors of CSBI in the past 6 months among MSM college students.

Variables	CSBI (*n* = 317) % (*n*/*n*)	Crude OR (95% CI)	*P*	Adjusted OR (95% CI)	*P*
Age, years					
18–20	41.7 (90/216)	1		1	
21–23	50.0 (179/358)	0.729 (0.451–1.180)	0.198	1.302 (0.908–1.868)	0.151
≥24	49.5 (48/97)	1.021 (0.652–1.599)	0.928	1.150 (0.689–1.918)	0.592
Registered permanent residence					
Zhejiang Province	44.6 (197/442)	1		–	–
Other Provinces	52.4 (120/229)	1.369 (0.994–1.886)	0.054	–	–
Educational level				–	–
Junior college	45.6 (94/206)			–	–
Bachelor’s degree or above	48.0 (223/465)	1.098 (0.790–1.526)	0.310	–	–
Disclosure of sexual orientation					
No	49.6 (141/284)	1		–	–
Yes	45.5 (176/387)	0.846 (0.622–1.150)	0.285	–	–
Frequency of watching pornography (times per month)					
<1 or never	34.8 (57/164)	1		1	
1–2	44.3 (66/149)	1.493 (0.946–2.355)	0.085	1.678 (1.040–2.708)	0.034
≥3	54.2 (194/358)	2.221 (1.514–3.256)	<0.001	2.404 (1.598–3.617)	<0.001
Sexual orientation					
Gay	48.0 (236/492)	1		1	
Bisexual or uncertain	45.3 (81/179)	0.897 (0.636–1.264)	0.533	0.922 (0.641–1.328)	0.663
Anal sexual role					
Mainly receptive	47.2 (136/288)	1		–	–
Mainly insertive	51.9 (107/206)	1.208 (0.844–1.728)	0.301	–	–
Both equally	41.8 (74/177)	0.803 (0.550–1.172)	0.255	–	–
Having a stable male sexual partner					
No	40.7 (166/408)	1		1	
Yes	57.4 (151/263)	1.965 (1.435–2.691)	<0.001	1.900 (1.369–2.637)	<0.001
HIV knowledge score					
0–4	46.1 (259/562)	1		–	–
5	53.2 (58/109)	1.330 (0.882–2.007)	0.174	–	–
Obtained HIV knowledge from MSM communities					
No	44.9 (71/158)	1		–	–
Yes	48.0 (246/513)	1.129 (0.784–1.165)	0.016	–	–
Previous PEP use					
No	45.9 (280/610)	1		1	
Yes	60.7 (37/61)	1.817 (1.061–3.111)	0.030	2.154 (1.220–3.802)	0.008
Heard of PrEP previously					
No	40.4 (42/104)	1		–	–
Yes	48.5 (275/567)	1.390 (0.909–2.126)	0.129	–	–
Previous HIV self-testing^a^					
No	40.1 (71/177)	1		1	
Yes	48.7 (232/476)	1.420 (1.000–2.015)	0.050	1.119 (0.771–1.624)	0.555

^a^Missing data for 18 participants who do not response for HIV self-testing.

### Characteristics of casual sexual behavior on the internet and inconsistent condom use

Of the 317 MSM college students who engaged in CSBI in the past 6 months, 270 (85.2%) reported having 1–3 CSPI during the period. Participants engaged in CSBI in specific settings: 28.1% (89/317) only on campus, 41.0% (130/317) only off campus, and 30.9% (98/317) both on and off campus. Additionally, 53.0% (168/317) took the receptive anal role during their most recent sexual encounter with a CSPI. Among MSM who found partners online, 31.5% (100/317) reported inconsistent condom use with CSPI in the past 6 months. Furthermore, 30.6% (97/317) reported stimulant use during the most recent sexual encounter with a CSPI, while 66.6% (211/317) reported inquiry into the HIV status of the most recent CSPI (Table 4).

**Table 4:** Type of sexual partners, condom use, and stimulant use related to engaging in CSBI in the past 6 months among MSM college students by sexual orientation (*n* = 317).

Variables	Total (*n* = 317) *n* (%)	Gay men (*n* = 236) *n* (%)	Bisexual or uncertain (*n* = 81) *n* (%)	*χ* ^2^	*P*
Number of CSPI in the past 6 months				0.520	0.471
1–3	270 (85.2)	203 (86.0)	67 (82.7)		
≥4	47 (14.8)	33 (14.0)	14 (17.3)		
Type of CSPI in the past 6 months				3.854	0.146
All students	89 (28.1)	72 (30.5)	17 (21.0)		
No students	130 (41.0)	97 (41.1)	33 (40.7)		
Both	98 (30.9)	67 (28.4)	31 (38.3)		
Anal sexual role during the most recent sex encounter with CSPI				19.075	<0.001
Receptive	168 (53.0)	142 (60.2)	26 (32.1)		
Insertive	149 (47.0)	94 (39.8)	55 (67.9)		
Condom use with CSPI in the past 6 months				9.344	0.009
Every time	217 (68.5)	172 (72.9)	45 (55.6)		
Mostly or rarely	72 (22.7)	48 (20.3)	24 (29.6)		
Never	28 (8.8)	16 (6.8)	12 (14.8)		
Stimulant use during the most recent sexual encounter with a CSPI				5.269	0.022
No	220 (69.4)	172 (72.9)	48 (59.3)		
Yes	97 (30.6)	64 (27.1)	33 (40.7)		
Inquiry into the HIV status of the most recent CSPI				1.927	0.165
No	106 (33.4)	84 (35.6)	22 (27.2)		
Yes	211 (66.6)	152 (64.4)	59 (72.8)		

Compared with gay men, participants who identified as bisexual or of uncertain sexual orientation reported higher proportion of insertive sexual role during the most recent sexual encounter with CSPI (67.9%, 55/81 vs. 39.8%, 94/236, *χ*^2^ = 19.075, *p* < 0.001) and stimulant use during the most recent sexual encounter with a CSPI (40.7%, 33/81 vs. 27.1%, 64/236, *χ*^2^ = 5.296, *p* = 0.022). The two groups also reported different proportion of condom use with CSPI in the past 6 months: “used condoms every time” (55.6%, 45/81 vs. 72.9%, 172/236), “used condoms most of the time or rarely” (29.6%, 24/81 vs. 20.3%, 48/236), “never used condoms” (14.8%, 12/81 *vs*. 6.8%, 16/236), *χ*^2^ = 9.344, *p* = 0 0.009 (Table 4).

### Associated factors of inconsistent condom use

In the multivariable logistic regression analyses, several correlates were associated with inconsistent condom use with CSPI in the past 6 months. Compared with participants who did not disclose their sexual orientation, those who disclosed their sexual orientation reported higher odds of inconsistent condom use (aOR = 1.711,95% CI: 1.010–2.898). Compared with gay men, individuals identifying as bisexual or uncertain sexual orientation reported higher odds of inconsistent use (aOR = 2.228, 95% CI: 1.241–3.999). Additionally, participants with casual sexual partners who were not solely students showed greater odds of inconsistent condom use than those whose casual partners were exclusively students (aOR = 1.897, 95% CI: 1.092–3.297). Meanwhile, compared with participants with low condom use self-efficacy, those with high condom use self-efficacy had lower odds of inconsistent condom use (aOR = 0.374, 95% CI: 0.212–0.657). Participants obtaining HIV knowledge from the MSM communities had lower odds of inconsistent condom use (aOR = 0.447, 95% CI: 0.245–0.815) compared with those not obtaining HIV knowledge from this source (Table 5).

**Table 5:** Univariate and multivariable logistic regression analyses for predictors of inconsistent condom use with CSPI in the past 6 months among MSM college students.

Variables	Inconsistent condom use (*n* = 100) % (*n*/*n*)	Crude OR (95% CI)	*P*	Adjusted OR (95% CI)	*P*
Age, years					
18–20	35.6 (32/90)	1		1	
21–23	30.2 (54/179)	1.340 (0.628–2.858)	0.449	0.693 (0.387–1.240)	0.216
≥24	29.2 (14/48)	1.049 (0.521–2.112)	0.893	0.823 (0.367–1.845)	0.637
Educational level					
Junior college	31.9 (30/94)	1		–	–
Bachelor or master	31.4 (70/223)	0.976 (0.582–1.638)	0.927	–	–
Disclosure of sexual orientation					
No	26.2 (37/141)	1		1	
Yes	35.8 (63/176)	1.567 (0.964–2.547)	0.070	1.711 (1.010–2.898)	0.046
Sexual orientation					
Gay	27.1 (64/236)	1		1	
Bisexual or uncertain	44.4 (36/81)	2.150 (1.273–3.630)	0.004	2.228 (1.241–3.999)	0.007
Anal sex role during the recent sexual encounter with CSPI					
Receptive	31.5 (53/168)	1		1	
Insertive	31.5 (47/149)	1.000 (0.622–1.607)	0.999	0.870 (0.508–1.490)	0.611
Types of CSPI in the past 6 months					
Students only	23.8 (31/130)	1		1	
Not just students	36.9 (69/187)	1.867 (1.132–3.082)	0.015	1.897 (1.092–3.297)	0.023
Stimulant use during the most recent sexual encounter with a CSPI					
No	28.6 (63/220)	1		1	
Yes	38.1 (37/97)	1.537 (0.929–2.542)	0.094	1.023 (0.582–1.800)	0.937
Inquiry into the HIV status of the most recent CSPI					
No	31.8 (67/211)	1		–	–
Yes	31.1 (33/106)	0.972 (0.588–1.607)	0.911	–	–
Condom use self-efficacy					
Low	49.4 (40/81)	1		1	
High	25.4 (60/236)	0.349 (0.207–0.591)	<0.001	0.374 (0.212–0.657)	0.001
Discussed condom use with peers					
No	37.5 (27/72)	1		–	–
Yes	29.8 (73/245)	0.707 (0.408–1.226)	0.217	–	–
HIV knowledge score					
0–4	32.8 (85/259)	1		–	–
5	25.9 (15/58)	0.714 (0.376–1.358)	0.304	–	–
Obtained HIV Knowledge from MSM communities					
No	43.7 (31/71)	1		1	
Yes	28.0 (69/246)	0.503 (0.292–0.868)	0.014	0.447 (0.245–0.815)	0.009
Previous PEP use					
No	31.1 (87/280)	1		1	
Yes	35.1 (13/37)	1.202 (0.584–2.471)	0.617	0.991 (0.452–2.171)	0.982
Heard of PrEP previously					
No	33.3 (14/42)	1		–	–
Yes	31.3 (86/275)	0.910 (0.456–1.815)	0.789	–	–
Previous HIV self-testing^b^					
No	28.2 (20/71)	1		–	–
Yes	32.3 (75/232)	1.218 (0.678–2.188)	0.509	–	–

^b^Missing data for 14 participants who do not response for HIV self-testing.

## Discussion

### Principal findings

Our goal was to draw attention to web-based casual sexual behavior and consistent condom use among MSM college students, as well as the differences in these risky behaviors between gay men and those identified as bisexual or other sexual orientations.

According to our data, nearly half of MSM college students reported engaging in CSBI in the past 6 months. The proportion was higher than that of non-student MSM in Zhejiang but lower than that reported in other countries (6, 10). However, the proportion of MSM college students who consistently used condoms with CSPI was comparable to that of non-student MSM (6). This finding may be related to their relatively fewer opportunities to acquire casual sexual partners and greater emphasis on privacy protection. The prevalence of MSM social app usage among college-attending MSM was as high as 98.2% (13). Compared with non-student MSM, MSM college students reported a higher prevalence of depression, which may be attributed to lower social support and a higher level of loneliness (19). Their heightened demand for confidentiality might be associated with frequent social app use. Furthermore, owing to our sampling strategy, recruited participants may possessed stronger social connections, greater health awareness, or higher levels of online engagement.

Exploring the correlates of CSBI and inconsistent condom use with CSPI is critical for HIV prevention among MSM, especially MSM college students. In this study, having a stable male sexual partner, PEP uptake, and frequent viewing of pornographic videos were independently associated with CSBI.

The higher likelihood of MSM with stable sexual partners engaging in CSBI might be the result of the combined effects of multiple factors: greater outness, pursuit of sexual freedom, longing for intimate relationships, and the high efficiency of the Internet (9, 19, 20). It represented a “rational choice” made by individuals in a specific social context—one that not only meets their sexual needs and identity recognition but also minimizes negative impacts on their stable relationships and personal lives.

The PEP was effective in preventing HIV infection among MSM (21, 22). In 2020, the National Medical Products Administration of China approved oral FTC/TDF for HIV prevention. In this study, only 10% of the participants reported having taken PEP, a proportion comparable to that reported in another study conducted in China (3.3%) (23). In Zhejiang, designated hospitals in each county-level administrative region provided PEP services. We hypothesized a bidirectional association between PEP uptake and risky sexual behaviors. Indeed, we identified a correlation between PEP uptake and CSBI. However, the direction of the association could not be determined due to the cross-sectional study design, and further research was required to elucidate the underlying causal relationship.

The PrEP was also an effective measure for HIV prevention (24). However, no significant association was observed between having heard of PrEP, engaging in CSBI and inconsistent condom use with CSPI in this study. In our province, the accessibility of PrEP services remained low, which precluded an in-depth analysis of PrEP in the present study. This factor should be incorporated into future research once PrEP services become more widely available in our province.

In this study, we hypothesized that MSM college students who watch pornographic videos frequently would be more prone to seeking sexual partners online. The results revealed that frequent viewing of pornographic videos (defined as three times or more per week) was positively associated with CSBI. Sexual desire might serve as an intermediate variable. The arousal of sexual desire is related to multiple factors. Research has showed strong correlations between cybersex addiction symptoms and indicators of sexual arousal and sexual excitability, coping strategies via sexual behaviors, and psychological symptoms (25). Individuals who frequently viewed pornographic videos require greater attention and targeted sexual education interventions.

Our findings indicated that bisexual participants and those with uncertain sexual orientation were more likely to report inconsistent condom use and stimulant use compared with gay men; however, no association was observed between sexual orientation and engaging in CSBI. The difficulty reported by bisexual men in refusing unprotected sexual behavior might be linked to discrimination within the gay community (26). Research has shown that bisexual men have a higher prevalence of psychological distress, such as depression (27, 28). A meta-analysis showed that the HIV prevalence among bisexual men was comparable to that among gay men (29). The high prevalence of HIV and risky behaviors among bisexual men elevated the risk of HIV transmission to their female sexual partners. However, few HIV prevention programs were tailored to address the unique needs of bisexual men (30). Therefore, MSM college students identified as bisexual or uncertain sexual orientation should not be overlooked and warrant further in-depth research.

The results showed that almost half of MSM college students had disclosed their sexual orientation. A study conducted in 13 European cities reported that 71% of MSM had disclosed their sexual orientation (31), a rate nearly 1.5 times that observed in the present study. Non-disclosure of sexual orientation could be a self-protective act to prevent negative reactions, but could lead to other adverse consequences (32, 33). Disclosure of sexual orientation may facilitate access to greater social support, yet it may also give rise to certain problems. Our study showed that those who disclosed their sexual orientation had significantly higher odds of inconsistent condom use. A study conducted in China among 295 gay and bisexual men found that non-disclosers were more likely than disclosers to report unprotected anal intercourse with men (aOR = 2.49) in the past 6 months (34). Therefore, further research should be conducted to explore this causal relationship, with adjustment of confounding factors such as social and cultural factors.

For MSM, Internet-based interventions have recently gained popularity and been shown to reduce HIV-related risk behaviors among a sample of gay and bisexual men living with HIV (35, 36). In this study, obtaining HIV knowledge from the MSM community was positively associated with consistent condom use with CSPI, but no association was found with the act of seeking such partners on the Internet. BA part from campus health education, MSM communities acted as the primary channel for MSM college students to acquire HIV prevention knowledge. Individuals who cannot access such knowledge from the MSM community may be socially isolated or indifferent to relevant information, which could lead to lower levels of condom use. However, the relationship between obtaining HIV knowledge from the MSM community and consistent condom use might be bidirectionally causal; cohort studies are therefore needed to infer a causal relationship. Strengthening MSM community support and providing HIV knowledge related to PEP, PrEP, HIV serosorting, and condom use might help reduce HIV-related risky behaviors (24, 37).

Participants who had sex with both student and non-student partners were less likely to use condoms. In a cohort study, the HIV incidence rate was 3.60 per 100 person-years among student MSM, which was lower than that among non-student MSM (5.88) (38). In Zhejiang Province, HIV transmission between students and non-student MSM had been confirmed via molecular transmission cluster analysis (39). Compared with non-student MSM, MSM college students had relatively limited sexual experience and insufficient sexual health knowledge (40). When engaging in sexual behavior with non-student MSM, they often adopt a receptive sexual role. Consequently, they were at a disadvantage in terms of communication and decision-making power regarding condom use. Promoting awareness and rejection of risky behaviors (e.g., sexual contact with non-student partners) among MSM college students should be a key target of future risk reduction programs.

We also hypothesized that inquiry into the HIV status of the most recent CSPI and discussion about condoms with friends might be related to condom use, which was not observed in this study. It could be an attribute to sampling and require further investigation.

### Limitations

The findings of this study should be interpreted in the context of its limitations. First, the cross-sectional design precludes the establishment of causal relationships. Second, the modified RDS method for recruitment methodology employed introduces potential sampling bias, which limits the generalizability of our finding to the broader population of socially active collegiate students MSM in Zhejiang province. Third, the research investigated sensitive sexual behaviors, and the primary outcomes were based on self-reports. This could result in information bias, social desirability bias, and recall bias. In addition, given the self-reported nature of all behavioral measurements, we should refrain from overinterpreting unmeasured behavioral mechanisms. Future studies should consider a cohort design and a probability random sampling to address the above drawbacks.

## Conclusion

A high frequency of online casual sexual behavior was observed among MSM college students, especially among those who frequently viewed pornography or maintained a stable male sexual partner. Participants identified as bisexual or uncertain sexual orientation, and having sexual contact with not solely students were more likely to report inconsistent condom use, a finding that highlights the need for targeted intervention measures. Additionally, MSM community-led interventions aimed at improving HIV knowledge and condom use self-efficacy could help promote consistent condom use with CSPI.

## Data Availability

The raw data supporting the conclusions of this article will be made available by the authors, without undue reservation.
